# Topical fluorouracil after surgery for ocular surface squamous neoplasia in Kenya: a randomised, double-blind, placebo-controlled trial

**DOI:** 10.1016/S2214-109X(16)30052-3

**Published:** 2016-05-17

**Authors:** Stephen Gichuhi, Ephantus Macharia, Joy Kabiru, Alain M'bongo Zindamoyen, Hilary Rono, Ernest Ollando, Joseph Wachira, Rhoda Munene, John Maina, Timothy Onyuma, Mandeep S Sagoo, Helen A Weiss, Matthew J Burton

**Affiliations:** a London School of Hygiene & Tropical Medicine, London, UK; b Department of Ophthalmology, University of Nairobi, Nairobi, Kenya; c PCEA Kikuyu Eye Unit, Kikuyu, Kenya; d Kitale District Hospital, Kitale, Kenya; e Sabatia Eye Hospital, Wodanga, Kenya; f Kenyatta National Hospital, Nairobi, Kenya; g UHEAL Foundation, Nairobi, Kenya; h Department of Pathology, MP Shah Hospital, Nairobi, Kenya; i UCL Institute of Ophthalmology, University College London, London, UK; j Moorfields Eye Hospital, London, UK; k St Bartholomew's Hospital, London, London, UK

## Abstract

**Background:**

Ocular surface squamous neoplasia (OSSN) is an aggressive eye tumour particularly affecting people with HIV in Africa. Primary treatment is surgical excision; however, tumour recurrence is common. We assessed the effect of fluorouracil 1% eye drops after surgery on recurrence.

**Methods:**

We did this multicentre, randomised, placebo-controlled trial in four centres in Kenya. We enrolled patients with histologically proven OSSN aged at least 18 years. After standard surgical excision, participants were randomly allocated to receive either topical fluorouracil 1% or placebo four times a day for 4 weeks. Randomisation was stratified by surgeon, and participants and trial personnel were masked to assignment. Patients were followed up at 1 month, 3 months, 6 months, and 12 months. The primary outcome was clinical recurrence (supported by histological assessment where available) by 1 year, and analysed by intention to treat. The sample size was recalculated because events were more common than anticipated, and trial enrolment was stopped early. The trial was registered with Pan-African Clinical Trials Registry (PACTR201207000396219).

**Findings:**

Between August, 2012, and July, 2014, we assigned 49 participants to fluorouracil and 49 to placebo. Four participants were lost to follow-up. Recurrences occurred in five (11%) of 47 patients in the fluorouracil group and 17 (36%) of 47 in the placebo group (odds ratio 0·21, 95% CI 0·07–0·63; p=0·01). Adjusting for passive smoking and antiretroviral therapy had little effect (odds ratio 0·23; 95% CI 0·07–0·75; p=0·02). Adverse effects occurred more commonly in the fluorouracil group, although they were transient and mild. Ocular discomfort occurred in 43 of 49 patients in the fluorouracil group versus 36 of 49 in the placebo group, epiphora occurred in 24 versus five, and eyelid skin inflammation occurred in seven versus none.

**Interpretation:**

Topical fluorouracil after surgery substantially reduced recurrence of OSSN, was well-tolerated, and its use recommended.

**Funding:**

British Council for Prevention of Blindness and the Wellcome Trust.

## Introduction

**Figure 1:**
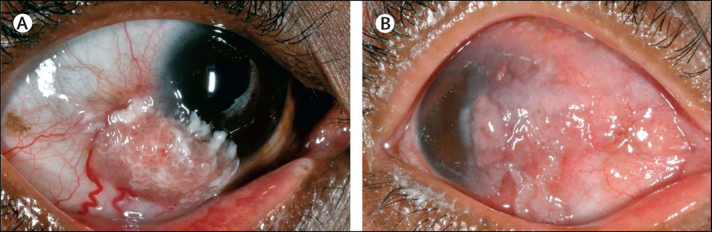
Ocular surface squamous neoplasia Moderately differentiated conjunctival squamous cell carcinoma, (A) moderate size, (B) large lesion involving the cornea, limbus, and extending to the fornix. Fornix involvement is often associated with orbital spread.

Ocular surface squamous neoplasia (OSSN) covers a range of conjunctival and corneal diseases, from intra-epithelial dysplasia to invasive squamous cell carcinoma.^1^ Risk factors for OSSN include ultraviolet radiation, HIV infection, and human papillomavirus infection.^2^ In temperate regions, OSSN is uncommon, usually growing slowly, and most often affects elderly men. By contrast, in sub-Saharan Africa, OSSN is more common and aggressive.^3^ It affects younger adults, predominately women (around two-thirds of cases), and is strongly associated with HIV infection (in about 70% of cases). OSSN has a wide range of clinical phenotypes and late presentation with invasive orbital disease is not uncommon (figure 1). Surgical excision is the mainstay of treatment, although primary chemotherapy has also been used (appendix). OSSN often recurs after surgery. The highest recorded recurrence is 67%.^4^ Recurrence is a particular problem in sub-Saharan Africa, where it typically occurs in 30–40% of patients.^4^, ^5^, ^6^, ^7^ In temperate climates, recurrence typically occurs in 5–25% of patients (appendix). Surgical outcomes seem to be affected by delays in diagnosis, tumour size, histological grade, ocular location, scleral invasion, excision margin involvement, prior excision, and coexisting xeroderma pigmentosum.^5^, ^8^, ^9^ Several adjunctive treatment regimens are used during or after surgery to reduce recurrence: cryotherapy, topical chemotherapy (mitomycin and fluorouracil), interferon alfa-2b, retinoic acid, and radiotherapy (appendix p 1, 3, 7, 11, 13, 14). Most data on adjuvant treatment are case series. There is one previous randomised trial, from Australia, which assessed the effectiveness of topical mitomycin and there are no trial data on interventions for people with HIV.^10^, ^11^ Radical surgery (enucleation or exenteration) is usually needed for advanced disease.^12^

The antimetabolite fluorouracil is often used in ophthalmology, particularly for its anti-scarring properties during surgical procedures (trabeculectomy, pterygium excision, and lacrimal surgery).^13^ Eye drops containing 1% fluorouracil have also been used for several years to treat patients with OSSN after tumour excision (appendix p 11), on the basis of case series, which suggest that fluorouracil reduces tumour recurrence and is safe.^14^, ^15^, ^16^, ^17^, ^18^, ^19^, ^20^ However, there are no data from trials. Fluorouracil is widely available and relatively cheap in sub-Saharan Africa, therefore, if shown to be an effective adjuvant, it would be a deliverable intervention.

**Research in context**

**Evidence before this study**

Ocular surface squamous neoplasia (OSSN) is an eye cancer, common in people with HIV. A Cochrane systematic review from 2013 showed no evidence from trials for the effectiveness of interventions used in this population. We searched electronic databases (PubMed, Embase, The Cochrane Library), clinical trial registries (WHO International Clinical Trials Registry Platform and the US National Institutes of Health ClinicalTrials.gov), and international conference proceedings of HIV/AIDS and AIDS-related cancers from the AIDS Education Global Education System for studies published up to Aug 31, 2015, irrespective of language or publication status. We used the terms “randomized controlled trial”, “controlled clinical trial”, “randomized”, “placebo”, “drug therapy”, “randomly”, “trial”, “conjunctiva*”, “ocular surface”, “carcinoma”, “cancer”, “neoplasia”, “neoplasm”, “neoplastic”, “dysplasia”, “dysplastic”, “squamous”, and “squamous cell”. We found only one trial, on topical mitomycin in a non-HIV-infected population in Australia. We identified some case series and case reports (appendix). Only series that reported recurrence as an outcome were included.

**Added value of this study**

Our study provides the first evidence from a trial of the effectiveness of fluorouracil as adjunctive treatment for OSSN. Our results show that the simple and relatively inexpensive use of 4 weeks of fluorouracil 1% eye drops after surgical excision substantially reduced the relative risk of recurrence compared with placebo and was safe. There were transient side-effects, such as watery eye, discomfort when taking the drops, and eyelid skin inflammation but these were mostly mild and resolved in a few weeks after completion of 4 week treatment.

**Implications of all the available evidence**

Recurrence is a huge issue in the management of this common and aggressive eye disease. Fluorouracil does not need stringent storage conditions and cytotoxics have a low risk of contamination. It is on the WHO Essential Medicines List, and is a widely available and low-cost option, particularly in sub-Saharan Africa, which has the highest incidence of OSSN in the world. Translation of these trial results into clinical practice is therefore feasible.

We assessed whether use of fluorouracil 1% eye drops could reduce recurrence of OSSN following surgical excision in Kenya.

## Methods

### Study design and participants

We did a double-blind, parallel-group, randomised, placebo-controlled trial at four centres in Kenya: Kenyatta National Hospital Eye Clinic in Nairobi, PCEA Kikuyu Eye Unit in central Kenya, Sabatia Eye Hospital in western Kenya, and Kitale District Hospital in the north Rift Valley.

We enrolled consecutive patients presenting with suspicious conjunctival lesions. Entry criteria were: histologically proven OSSN involving two or fewer quadrants; attendance for follow-up within the first 2 months after excision; healing of the excision site; and age at least 18 years. Exclusion criteria were: previous treatment with topical antimetabolite drugs such as fluorouracil or mitomycin to the same eye or systemic cytotoxic drugs; extensive disease requiring more radical surgery than a simple excision; and pregnant or breastfeeding mothers. Patients were not enrolled if they did not think that they could return for follow-up.

All participants gave written informed consent before enrolment. Ethics approval was granted by the Kenyatta National Hospital/University of Nairobi ethics and research committee and the London School of Hygiene & Tropical Medicine ethics committee. Approval was also obtained from the Kenya Pharmacy and Poisons Board to produce and use the active intervention drops because they are not commercially available.

An independent data and safety monitoring board oversaw the study, confirmed data integrity, and approved the results and report for release. Trial personnel received good clinical practice training and certification. This study adhered to the tenets of the Declaration of Helsinki.

### Randomisation and masking

Participants were randomly assigned (1:1) to either fluorouracil 1% or placebo eye drops. The fluorouracil eye drops were prepared by dilution of fluorouracil 50 g/L solution for injection in hydroxypropyl methylcellulose 0·7% artificial tear eye drops. The placebo was the same hydroxypropyl methylcellulose 0·7% artificial tear eye drops.

The randomisation sequence was generated by computer by the trial statistician using Stata (version 12). The permuted block size (known only to the statistician) varied randomly between two and four, and randomisation was stratified by surgeon. The allocation sequence was transferred to the manufacturing pharmacy, where an independent pharmacist applied labels with sequential code numbers to the appropriate eye drop bottles. Participants, clinicians, and study personnel were masked to the allocation: the bottles, liquid content, and packaging had identical appearances. The supervising clinician at each of the four study centres issued the trial drug to participants. The allocation followed the order of enrolment.

### Procedures

Lesions involving two or fewer quadrants of the conjunctiva were fully excised with a 4 mm clear margin by the no-touch technique, with use of an operating microscope and under local anaesthesia.^21^ Absolute alcohol was applied to any corneal component of the tumour to loosen it and facilitate dissection. The conjunctival component was dissected down to bare sclera. Cryotherapy was not applied, because it is not generally available in sub-Saharan Africa. Topical adrenaline, and where necessary mild diathermy, were used for haemostasis. The conjunctiva around the defect was undermined and mobilised for primary closure. Specimens were placed on suture-pack polystyrene, to keep the tissue flat and oriented for the pathologist, and fixed in 10% neutral buffered formalin. All histopathological tests were done centrally and reported by a single pathologist. Combined gentamicin 0·3% and prednisolone acetate 1% eye drops were applied four times per day for 3–4 weeks after surgery. Patients were reviewed after about 4 weeks to confirm wound healing and for recruitment into the trial.

Participants were asked to self-administer one drop of their allocated medication four times a day to the affected eye for 4 weeks. Each participant was given a 28 day medication diary to monitor treatment. They were asked to record each dose taken or missed. The record card had a similar diary for adverse effects (pain or burning sensation, excessive tears, and redness).

Participants underwent a detailed ophthalmic examination with a slit-lamp biomicroscope before surgery and at about 1 month after surgery. After enrolment, follow-up visits were scheduled for 1 month, 3 months, 6 months, and 12 months after randomisation. Participants were telephoned 1 week before their appointments to remind them. Individuals who missed follow-up visits were contacted by telephone. At each follow-up visit, a symptom history was taken and a detailed ophthalmic examination was done for evidence of recurrent disease. On each examination, high-resolution digital images of the surface of the eyes were taken. In addition, we assessed whether the lacrimal drainage system was blocked using the dye disappearance test. Fluorescein dye was applied in both eyes in the inferior conjunctival fornix and the tear film observed with the cobalt blue light of the slit lamp after 5 min for clearance of the dye. The presence of dye after this time was considered positive, indicating a functional or anatomical blockage.

HIV status was initially tested by ELISA using Vironostika antigen/antibody kit (Biomerieux, France) then later changed to rapid tests with Alere Determine HIV-1/2 Ag/Ab (Alere, USA) and Unigold (Trinity Biotech, USA). CD4 count was measured with FacsCount (Becton Dickinson, USA). Serum retinol concentration was quantified by high-performance liquid chromatography (SHIMADZU Prominence HCT2010, Japan).

When an obvious regrowth was found, re-excision for treatment and histopathology was advised. If a small potentially suspicious change was observed, it was initially photographed, the size measured, and the participant examined more frequently than the scheduled study visits. If on subsequent visits the lesion had progressed, the lesion was re-excised and sent for histopathology; the date of recurrence was recorded as the first time the possible regrowing lesion was noticed.

### Outcomes

The primary outcome was clinical recurrence of the lesion at any time during the first year of follow-up, confirmed by histological assessment where available. The secondary outcomes were time to recurrence, cofactors of recurrence, and adverse events.

Primary outcome events were assessed and confirmed centrally by an ophthalmologist, who had either directly examined all patients at Kenyatta National Hospital and Kikuyu Eye Unit, or reviewed the clinical images from Sabatia Eye Hospital and Kitale District Hospital, supported by histopathological results. For cases where histopathology was not available, mostly because the participant did not return for the repeat surgery, the images of recurrent lesions were reviewed by two consultant ophthalmologists experienced in OSSN in east Africa to confirm clinical recurrence. Adverse effects were monitored by reviewing the medication diary with the participant and asking about discomfort and tearing.

### Statistical analysis

Case series of the use of surgical excision with or without adjuvant fluorouracil treatment have reported recurrences in 3·2–43% of patients.^7^, ^19^, ^22^, ^23^ Assuming a recurrence of 20% in the control group and 10% in the treatment group, power of 80%, and a two-sided α of 5%, the target sample size was initially calculated to be 219 participants in each group. 1 year into the study, we noted that recruitment was slow but recurrences were more common overall than anticipated, so after review by the trial steering committee in discussion with the data safety and monitoring board, a pragmatic decision was made to revise the sample size assuming that 30% of patients in the placebo group and 5% in the treatment group would have disease recurrence. As such, a sample size of 43 participants in each group would provide 80% power to detect an absolute difference in recurrence rates of 25%.

The analysis was predefined. We compared the two groups for balance in terms of predefined factors that could have a bearing on aetiology or recurrence: age, sex, smoking history, outdoor occupation, HIV status, CD4 count, vitamin A concentration, tumour size, prior excision, and histological grade.^3^, ^5^, ^8^, ^9^ The primary analysis of the primary outcome and the safety analysis were done by intention to treat. Data were managed in Microsoft Access, cleaned, and transferred into Stata (version 12.1) for analysis.

We calculated the numbers of events, person-months, and rate of recurrence in each group. We estimated the effect size as the odds ratio (OR) for recurrence, estimated by logistic regression, with 95% CIs. We adjusted the crude OR for the seven surgeons as a random effect and for additional baseline factors that were greater in one group than the other.^24^ We analysed the effect of the intervention on time to recurrence with Kaplan-Meier survival curves, and we used Cox regression to estimate hazard ratios and 95% CIs, adjusting for substantial baseline imbalances. To assess whether survival was the same by treatment group, we used the log rank test. We report the risk of any adverse effects at any follow-up in the treated eye by group.

The trial is registered with the Pan-African Clinical Trials Registry, number PACTR201207000396219.

### Role of the funding source

The funders had no role in study design, data collection, data analysis, data interpretation, or writing of the report. The corresponding author had full access to all the data in the study and had final responsibility for the decision to submit for publication.

## Results

**Figure 2:**
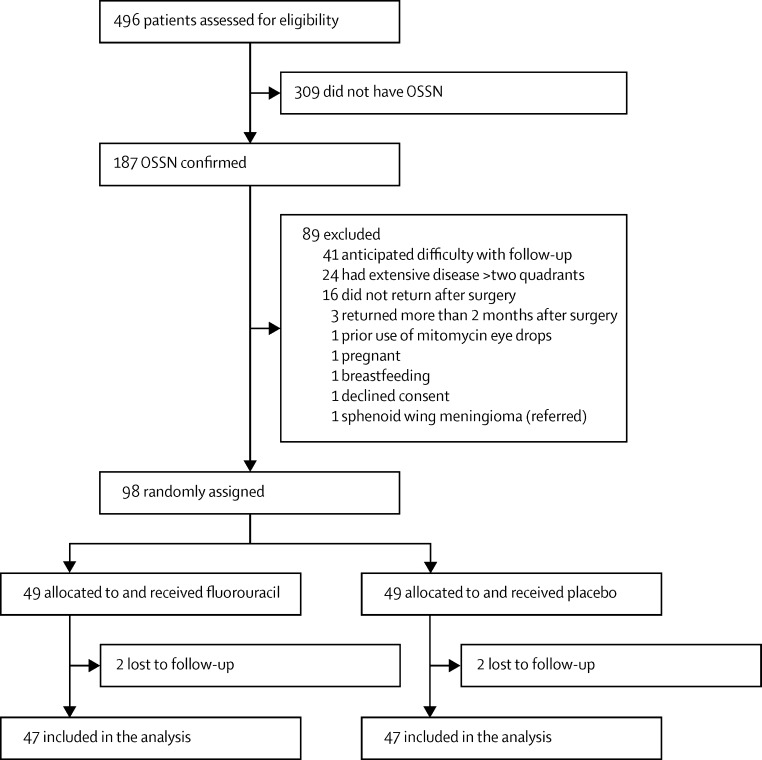
Trial profile OSSN=ocular surface squamous neoplasia.

Between August, 2012, and July 2014, 496 patients with conjunctival lesions had surgical excision followed by histopathological tests. 187 of these patients had OSSN and 309 had other pathological disease types. For all participants with OSSN, only one eye was involved. 89 (48%) of 187 patients were ineligible. Therefore, we enrolled 98 patients (figure 2). 49 patients were assigned to receive fluorouracil eye drops and 49 were assigned to placebo. 58 of 98 patients were enrolled at Kikuyu Eye Unit, 20 at Sabatia Eye Hospital, 12 at Kitale District Hospital, and eight at Kenyatta National Hospital. The final follow-up visits were scheduled for 12 months, although we included data from up to 13 months for late participants. Follow-up was completed in July, 2015. Four individuals without a recorded recurrence (two from each group) did not complete a full year of follow-up.

The main reasons for exclusion were: inability to return for regular follow-up (n=41), extensive disease requiring radical surgery (n=24), not returning at all after surgery (n=16), or returning more than 2 months after surgery (n=3; figure 2). There were no significant differences between the enrolled and non-enrolled patients in terms of age, sex, smoking, HIV infection, or stage of OSSN (excluding larger tumours; data not shown).

The mean age of all participants was 41·0 years (SD 11·3) and most were female. Baseline characteristics were reasonably balanced between the two groups with the exception of past passive cigarette smoking and use of antiretroviral therapy, which were more common in the placebo group than in the fluorouracil group, and therefore were adjusted for in the primary analysis (table 1).

By the end of follow-up, recurrent lesions had developed in 22 eyes of 22 participants. The four participants who did not complete a full year of follow-up were excluded from the primary analysis of clinical recurrence of the lesion by 1 year. Lesion recurrence was significantly less common in the fluorouracil group (five [11%] of 47 patients) than in the placebo group (17 [36%] of 47 patients; crude OR 0·21, 95% CI 0·07–0·63; p=0·01). This effect remained significant when adjusted for use of antiretroviral therapy and passive cigarette smoking (OR 0·23, 95% CI 0·07–0·75; p=0·02). The relative risk of recurrence was reduced by 70·7% and the absolute difference was 25·6%. Treatment with fluorouracil after surgery for four patients would therefore prevent an estimated one recurrence (number needed to treat 3·9, 95% CI 2·4–11·8).

16 of 22 recurrent lesions underwent repeated surgical excision and recurrent OSSN was confirmed by histopathology in all cases. Re-excision was not done for six recurrent cases: four participants in the placebo group did not return for repeat surgery or further follow-up after the recurrence was noted, and two participants in the placebo group died before re-excision could be done (one from a presumed myocardial infarction and one from HIV-related complications). The images of these cases were reviewed by two ophthalmologists experienced in OSSN in east Africa. All were judged to be recurrent OSSN disease on clinical grounds, in the context of previous histologically confirmed OSSN.

**Figure 3:**
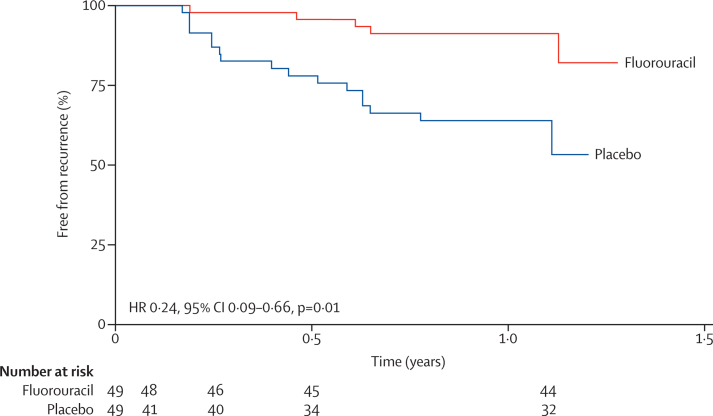
Kaplan-Meier analysis of time to recurrence

There was a significant difference in the recurrence rate between the two groups over the follow-up period (hazard ratio [HR] 0·24, 95% CI 0·09–0·66; p=0·01; figure 3), which changed slightly after adjusting for smoking and use of antiretroviral therapy (HR 0·32, 95% CI 0·11–0·95; p=0·04). The test for proportional hazards assumption showed that the assumption of proportionality was appropriate (p=0·59). Median tumour-free survival was 7·3 months (IQR 2·3–13·5) in the fluorouracil group and 4·8 months (3·0–7·6) in the placebo group but the difference was not statistically significant (p=0·23).

Sensitivity analysis, assuming the four participants who did not complete 1 year of follow-up did not have a recurrence in the first year, made little difference to the results (crude OR 0·21, 95% CI 0·07–0·64, p=0·01; adjusted OR 0·25, 95% CI 0·08–0·79, p=0·02; HR 0·25, 95% CI 0·07–0·67, p=0·01; adjusted HR 0·33, 95% CI 0·11–0·97, p=0·04) and the number needed to treat remained at four (4·1, 95% CI 2·5–12·5).

Tumour size at baseline was a significant cofactor of recurrence (crude OR 1·27, 95% CI 1·04–1·54, p=0·02). Participants who had a recurrence had significantly larger mean tumour diameter (7·3 mm, SE 0·26) than did patients who did not have a recurrence (5·8 mm, SE 0·66; p=0·01). There was no effect modification by tumour size or rate of tumour growth before surgery, defined as tumour diameter divided by duration between noticing the growth and time of surgery, assuming a linear rate (likelihood ratio test p=0·49). The mean growth rate was 1·6 mm per month (SE 0·61) in patients who had a recurrence and 1·7 mm per month (SE 0·22) in patients who did not. Surgical margin involvement was not a significant cofactor for recurrence (crude OR 1·28, 95% CI 0·49–3·33, p=0·62) and nor was having invasive carcinoma rather than carcinoma in situ at baseline (crude OR 1·09, 95% CI 0·42–2·81, p=0·87).

Adverse effects were more common in the fluorouracil group than in the placebo group, as shown by analysis of the time to first adverse event (p=0·005; table 2). Epiphora (watery eye) was more common in the fluorouracil group than in the placebo group and the dye disappearance test became transiently positive in six patients in the fluorouracil group at 1 month. None of the participants with epiphora had a positive dye disappearance test at 1 year. Ocular discomfort was more common in the fluorouracil group than in the placebo group at 1 month (p=0·004). In one (2%) patient, discomfort was sufficient to discontinue treatment after 3 weeks. After the 1 month visit, no participants reported ocular discomfort.

Seven participants, all in the fluorouracil group, developed inflammation or irritation of the eyelid skin after about 2–3 weeks of treatment. This was attributed to overflow spillage of the eye drop onto the skin. All skin changes fully resolved within 1 month. We advised all further enrollees to apply drops while holding a piece of tissue paper against the lid; subsequently, no episodes of eyelid skin irritation occurred.

## Discussion

We showed that 4 weeks of treatment with topical fluorouracil 1% after surgical excision substantially reduced the 1 year recurrence of OSSN tumours. The study was done in a region with a relatively high incidence of OSSN, which is often associated with HIV infection, and where patients often present late with advanced disease. Tumour recurrence has been a major problem in managing this disease. Most of our participants were relatively young, and women outnumbered men, the typical demographic pattern in Africa.^3^ The whole range of OSSN disease was represented, enabling us to draw a general conclusion about the effectiveness of the intervention.

This study was the first randomised controlled trial of topical fluorouracil as adjunctive treatment for OSSN. However, our results are consistent with those of non-randomised case series of adjuvant fluorouracil, which have reported similarly low proportions of recurrence.^14^, ^15^, ^16^, ^17^, ^18^, ^19^, ^20^ Some investigators have reported the effectiveness of fluorouracil as primary treatment in presumed OSSN lesions without surgical excision and histopathological assessment.^14^ We chose to test fluorouracil in this setting because it is cheap and readily available with a history of use and wide acceptance for other types of ophthalmic surgery. Fluorouracil is on WHO's list of essential drugs. It does not require stringent storage conditions such as refrigeration. Therefore, the translation of this result into clinical practice, given the resource limitations of the Kenyan health system and other similar settings, is realistic. Because surgical cryotherapy is not routinely available, topical fluorouracil is therefore an alternative strategy to prevent recurrence.

The only other randomised study of treatment for OSSN was a placebo-controlled, crossover trial of topical mitomycin for 48 patients from Australia.^10^ However, that study has several distinct differences to our study, which probably limit the relevance to settings such as Kenya. First, only partial incisional biopsy samples were taken for diagnosis; the tumours were not excised. Given the advanced and aggressive disease in Africa, complete surgical removal of the lesion is the preferred approach. Second, the casemix was different: patients with squamous cell carcinoma were excluded from the Australia study, the population group was older than ours (mean age 67 years), predominantly male (75%), and probably not infected with HIV (no data were provided). Although the lesions regressed clinically on treatment with mitomycin, more than half of patients had persistent OSSN on repeat histological assessment of the lesion site 1 year after treatment.

Overall, the use of topical fluorouracil was associated with transient side-effects: watery eye, discomfort when taking the drops, and eyelid skin inflammation. However, these were mostly mild and resolved after the completion of treatment. A transiently positive dye disappearance test indicates temporary reversible obstruction of the nasolacrimal duct, a known complication of fluorouracil treatment.^18^ The most significant adverse effect was the eyelid skin inflammation. This was reliably prevented by protecting the skin with a tissue while applying the drops to catch any overflow. Epiphora was reported by 10% of participants at 1 month in the placebo group and 61% reported occasional discomfort at 1 month. We think that these effects were not caused by the placebo, which was a bland lubricant, but rather related to having recently had, often extensive, excision surgery to remove a tumour. It is quite common for excision of conjunctival lesions, OSSN or other pathology such as a pterygium, to result in a degree of ocular surface inflammation and irritation that can persist for several weeks. Such effects are especially common in young people of African origin, who are more likely to scar and have inflammation than are older white patients.^25^ Overall, we think that these side-effects can be partly mitigated, do not usually represent a problem after cessation of treatment, and are outweighed by the benefit of the reduced tumour recurrence.

Adjunctive fluorouracil probably works through its effect on residual OSSN cells that are left after surgical excision. It interferes with DNA and RNA processes through several active metabolites.^26^ Fluorodeoxyuridine monophosphate inhibits thymidylate synthase, blocking thymidylate production, and thus DNA replication. Rapidly dividing neoplastic cells are much more vulnerable to thymidine depletion than are normal cells. Fluorodeoxyuridine triphosphate is misincorporated into DNA and fluorouridine triphosphate is misincorporated into RNA. These different metabolites disrupt crucial cellular mechanisms, triggering apoptosis.

We were able to follow up participants to 1 year. This was attributable to two factors. First, we excluded people who said that they would be unlikely to return for follow-up. Second, the study team were careful to build and maintain good relationships with study participants, and actively communicated with those who missed follow-up visits.

Our study has several limitations. First, recruitment was slower than anticipated, resulting in a smaller study than originally anticipated. The initial study size was based on previously reported recurrence rates from several case series, with heterogeneous inclusion criteria, treatment regimens, and follow-up.^4^, ^7^, ^19^, ^22^, ^23^, ^27^, ^28^ However, the higher than expected recurrence rate and high retention enabled us to have good power with a smaller sample size. In common with other studies from Africa, the recurrence rate in both groups was higher than that reported in many case series from temperate countries. This difference is possibly because OSSN in this population, with a high proportion of patients who are HIV positive, is more aggressive and patients probably present later with more advanced disease.

Second, not all the cases of clinical recurrence had re-excision and histopathological tests done. However, all suspected recurrences for which histopathological results were available were confirmed as recurrent OSSN, which suggests that our clinical judgment in this situation is highly concordant with the pathology.

Third, we excluded a high proportion of potential participants. The most common reason for exclusion was that the patient was unlikely to return for follow-up. Excluding those who did not think they could return helped us achieve good follow-up among those who were enrolled in the trial. However, there was no systematic difference between participants and excluded patients in terms of age, sex, HIV status, smoking status, or OSSN grade (when those with large lesions requiring alternative radical surgery were excluded). This finding suggests that our results can be generalised. The participants who were lost to follow-up attended at least the first visit after randomisation and were recurrence-free at that point. The challenge of ensuring high follow-up rates in Kenyans with HIV has been reported previously.^29^

Fourth, we excluded individuals with very large tumours that required either enucleation or exenteration. This exclusion could reduce the generalisability of the findings. However, such patients are not suitable for less radical surgery and topical chemotherapy, as the tumour is already invading the deeper tissues of the orbit. Finally, there were some differences in adverse events by group, which could have led to unmasking. However, we think that this is unlikely: discomfort was common and similar in each group at 1 month, and eyelid inflammation, positive dye disappearance test, and epiphora after 1 month were uncommon.

In conclusion, 4 weeks of topical fluorouracil 1% after surgical excision of OSSN substantially reduced the 1 year recurrence of tumours. The treatment is safe, generally well tolerated, and easy to use. Fluorouracil is widely available, affordable, and easy to formulate into eye drops. It is suitable in settings without cryotherapy. Fluorouracil eye drops are an effective and realistic intervention to improve outcomes for people with OSSN.

## Figures and Tables

**Table 1:** Baseline characteristics

			**Fluorouracil group (n=49)**	**Placebo group (n=49)**
Sex				
	Male		17 (35%)	14 (29%)
	Female		32 (65%)	35 (71%)
Age (years)			39·1 (9·2)	42·9 (13·0)
Marital status*				
	Single		6 (12%)	11 (22%)
	Married		32 (65%)	28 (58%)
	Divorced or separated		4 (8%)	2 (4%)
	Widowed		7 (14%)	7 (14%)
Formal education (years)*				
	Tertiary (>12)		5 (10%)	4 (8%)
	Secondary completed (12)		18 (37%)	20 (41%)
	Some secondary (8–12)		4 (8%)	6 (12%)
	Primary completed (8)		13 (27%)	11 (22%)
	Some primary (<8)		6 (12%)	4 (8%)
	None		3 (6%)	3 (6%)
Past cigarette smoking*				
	No		41 (84%)	32 (67%)
	Yes		5 (10%)	4 (8%)
	Passive (spouse or partner smokes)		3 (6%)	12 (25%)
Current cigarette smoking†				
	No		44 (90%)	41 (87%)
	Yes		3 (6%)	0 (0%)
	Passive (spouse or partner smokes)		2 (4%)	6 (13%)
Cigarettes smoked daily			9 (6)	13 (13)
Years of cigarette smoking			10·7 (6·8)	16·2 (7·4)
Location of current occupation*				
	Indoors		20 (41%)	19 (40%)
	Outdoors		29 (59%)	29 (60%)
		Wears hat or cap outdoors†	8 (16%)	6 (13%)
		Wears sunglasses outdoors†	4 (8%)	4 (9%)
HIV infection‡			29 (63%)	31 (71%)
Use of antiretroviral therapy§			10 (22%)	19 (42%)
CD4 count (cells per μL)¶			444 (370)	460 (421)
Serum retinol concentration (μg/L)‖			489 (157)	529 (215)
Vitamin A deficiency (serum retinol <300 μg/L)‖			3 (6%)	3 (6%)
Tumour diameter (mm)			5·9 (2·6)	6·3 (2·4)
Prior excision			8 (16%)	9 (19%)
Histological grading of tumours				
	CIN 1		4 (8%)	4 (8%)
	CIN 2		13 (27%)	8 (16%)
	CIN 3		11 (22%)	14 (29%)
	Carcinoma in situ		0 (0%)	1 (2%)
	Poorly differentiated squamous cell carcinoma		1 (2%)	1 (2%)
	Moderately differentiated squamous cell carcinoma		17 (35%)	18 (37%)
	Well differentiated squamous cell carcinoma		3 (6%)	3 (6%)
Surgical margin involvement			21 (43%)	19 (39%)
Stage of OSSN**				
	T1N0M0		15 (31%)	9 (18%)
	T2N0M0		10 (20%)	9 (18%)
	T3N0M0		23 (47%)	31 (63%)
	T3N1M0		1 (2%)	0 (0%)

Data are n (%) or mean (SD).

* Data missing for one participant in the placebo group.

† Data missing for two participants in the placebo group.

‡ Data missing for three participants in the fluorouracil group and five in the placebo group.

§ Data missing for three participants in the fluorouracil group and four in the placebo group.

¶ Data missing for eight participants in the fluorouracil group and 11 in the placebo group.

‖ Data missing for ten participants in the fluorouracil group and 13 in the placebo group.

** As per the American Joint Committee on Cancer. CIN=cervical intra-epithelial neoplasia. OSSN=ocular surface squamous neoplasia.

**Table 2:** Adverse events

		**Fluorouracil group (n=49)**	**Placebo group (n=49)**	**p value**
Epiphora		..	..	..
	1 month	24 (49%)	5 (10%)	<0·001
	3 months*	3 (6%)	2 (4%)	0·66
	6 months†	1 (2%)	0 (0%)	0·32
	12 months†	4 (8%)	0 (0%)	0·04
Positive dye disappearance test		..	..	..
	At baseline	0 (0%)	1 (2%)	0·32
	1 month	6 (12%)	1 (2%)	0·05
	3 months*	1 (2%)	0 (0%)	0·32
	6 months†	0 (0%)	0 (0%)	-
	12 months†	0 (0%)	0 (0%)	-
Discomfort in the treated eye at 1 month‡		..	..	0·004
	Occasional discomfort	21 (43%)	30 (61%)	..
	Discomfort for <5 min	12 (24%)	3 (6%)	..
	Discomfort for ≥5 min	4 (8%)	2 (4%)	..
	Discomfort making treatment difficult	6 (12%)	1 (2%)	..
Eyelid inflammation at 1 month		7 (14%)	0 (0%)	<0·001
Any adverse event		..	..	0·005§
	1 month	34 (69%)	19 (39%)	..
	3 months	10 (20%)	9 (18%)	..
	6 months	5 (10%)	1 (2%)	..
	12 months	7 (14%)	6 (12%)	..

* 48 patients in the fluorouracil group and 47 in the placebo group because of loss to follow-up.

† 47 participants in each group because of loss to follow-up.

‡ No participant reported any discomfort at 3 months, 6 months, or 12 months.

§ Computed with the stratified log-rank test for equality of survivor functions.
