# Correction to *Lancet Glob Health* 2016; 4: e378–85

**DOI:** 10.1016/S2214-109X(17)30157-2

**Published:** 2017-04-07

*Gichuhi S, Macharia E, Kabiru J, et al. Topical fluorouracil after surgery for ocular surface squamous neoplasia in Kenya: a randomised, double-blind, placebo-controlled trial.* Lancet Glob Health *2016; **4:** e378–85*—In this Article, the definition of CIN in table 1 should have read “CIN=conjunctival intra-epithelial neoplasia”. This correction has been made to the online version as of April 7, 2017.

