# Supplementary material for: Topical fluorouracil after surgery for ocular surface squamous neoplasia in Kenya: a randomised, double-blind, placebo-controlled trial
Source: Lancet Glob Health. 2016 May 17;4(6):e378–85. doi: 10.1016/S2214-109X(16)30052-3 (PMC5081398; doi:10.1016/S2214-109X(16)30052-3)
Supplement: Supplementary appendix [file mmc1.pdf]

THE LANCET Global Health

Supplementary appendix

This appendix formed part of the original submission and has been peer reviewed. We post it as supplied by the authors.

Supplement to: Gichuhi S, Macharia E, Kabiru J, et al. Topical fluorouracil after surgery for ocular surface squamous neoplasia in Kenya: a randomised, double-blind, placebo-controlled trial. *Lancet Glob Health* 2016; **4:** e378–85.

Supplementary Table. Review of interventions used in ocular surface squamous neoplasia (OSSN)

A) Surgery ± Cryotherapy						
**Year**	**Country**	**Study size**	**Average age (years)**	**M:F ratio**	**Intervention**	**Recurrence**
2015	Tunisia[1]	79	61·1	2·56:1	excision 89·8%; margin excision + cryotherapy 46·6%; enucleation 2·5%; exenteration 6·4%	33·3% after 9 months
2015	USA[2]	43	68·4	7:3	excision + cryotherapy. Alcohol epitheliectomy for corneal extension. At surgeon's discretion AMT with sutures or glue	7·1% after 1year
2014	Germany[3]	38	70·6	1·4:1	excision (n=25). If complete excision was not feasible adjuvant IFN was used (n=14) applied one drop 5 times daily for 6 weeks. If the tumour invaded deep orbital structures exenteration was done (n=10)	52% of excisions and 20% of exenterations. Average time to recurrence 24 months
2014	USA[4]	98	64·0	1:1	excision alone (n=49) ; excision + cryotherapy (n=41) or intraoperative MMC (n=1) or sclerectomy (n=8) + AMT (n=14) or conjunctival autograft (n=1) or primary closure (n=10) or bare sclera (n=24) vs IFN drops (n=40)or subconj/perilesion IFN injection (n=1) or combined drops+injection (n=8). The dose was 3 million IU (in 0·5 ml) in all cases treated with subconjunctival injections. For topical therapy, the dose was 1 million IU/ml in 35 patients and 3 million IU/ml in 13 patients.	1% in the surgery group and 3% in the IFN group after 1yr
2013	Argentina[5]	4	53·2	1:1	excision + cryotherapy and primary closure	None after 6·5 years
2012	Iran[6]	17	70·7	7·5:1	excision + cryotherapy then Chloramphenicol/Betamethasone drops QID x 1wk; then MMC 0·04% x7-10 days;	5·9% after 9 months
2006	Uganda[7]	476	32·0	1:1·2	401 had eye-conserving excision, lamellar sclerectomy if deeply fixed, corneal dissection with a blade then wiped with alcohol, primary closure. No cryotherapy.	13 of the 401 (3·2%) after median follow up of 32 months
2003	Turkey[8]	57	55·0	1·9:1	excision + cryotherapy before and after the excision	12·3% after 31·7 months median follow up
2002	Mexico[9]	287	60·4	1·2:1	Local resection in 258 cases (90%), exenteration in 18 (6%), enucleation in 7 (2%) and radiation therapy in 2 (1%). One patient was treated with local resection and gamma irradiation, and another with local resection and application of cryotherapy at the surgical margins	5·2% after 7·7 months median follow up
2002	India[10]	5	?	?	Excision + cryotherapy + MMC	None at 1 year
2002	Australia[11]	26	65·1	3·3:1	Excision± cryotherapy (n=19), exenteration (n=6)	27% within 4-15 months
2000	USA[12]	28	66·6	4:1	19 had Excision alone (E) and 9 had excision+cryotherapy (E+C)	overall 5 (17·9%) recurred. 2 in the E+C and 3 in the E group. Mean follow up was 13months for primary lesions and 28months for recurrent lesions
1999	USA[13]	60	64·0	2·3:1	27excision+cryotherapy ; 14 excision+cryotherapy+sclerectomy; 8 excision+cryotherapy+mucosal graft; 1 exenteration; 1 enucleation	5% after a median of 56 months follow up
1997	Australia[14]	79	64·1	5:1	Excision	39% (31 eyes) overall recurred after a mean 4·6 years follow up
1989	Turkey[15]	22	?		Excision + cryotherapy before and after excision	9% (time point unclear)
1983	USA[16]	9	71·0	2:1	Excision + cryotherapy	22% after 9 months
**B) Surgery with amniotic membrane transplant (AMT)**						
**Year**	**Country**	**Study size**	**age (years)**	**M:F ratio**	**Intervention**	**Recurrence**
2014	Turkey[17]	21	62·4	1:2	AMT. no touch excision with 2mm margin + double freeze-thaw cryotherapy for 3 sec ± Alcohol epitheliectomy for corneal extension. The conjunctival defects were reconstructed up to the limbus with	None after 31 months follow up

					one sheet of cryotherapypreserved single layer AMT—the stromal side facing down—secured side to side with 8/0 vicryl sutures	
2011	Lithuania[18]	2			AMT	None after 38 months follow up
2008	Brazil[19]	8			AMT	1 (12·5%)recurred after 17·8 months mean follow up
2003	Taiwan[20]	5	64·6	3:2	excised with 3mm margin + AMT with basement membrane facing up.	None after mean 27 months follow up
2002	USA[21]	10	58·6		excised with a 3–4 mm lesion free margin and a superficial keratectomy was performed if there was corneal infiltration. AMT was placed over the surgical defect with the basement membrane side facing up and secured to the adjacent conjunctiva and episclera by interrupted or continuous 10-0 Vicryl sutures, making sure that its borders were placed under the conjunctival margin.	1 case (10%) with follow up period unclear

C) Immune-therapy with Interferon α 2b (IFNα2b)

Year	Country	Study size	age (years)	M:F ratio	intervention	Recurrence at median/mean follow up	Adverse effects
2014	Iran[22]	5	60·2	4:1	IFNα2b 3 million IU/ml QID x 4wks	None after median follow up 10·2 months	No patient developed persistent epithelial defect, symblepharon, limbal stem deficiency or systemic adverse effects related to IFNα2b therapy such as malaise, flu like symptoms, bone pain and fatigue.
2013	USA[23]	81	?	?	IFNα2b alone (n = 22, 27%) or combined with surgery (n = 59, 73%)	5% (4/81) of cases over a median follow-up of 1 year	Conjunctival hyperaemia (n = 4, 5%), ocular irritation (n = 3, 4%), superficial punctate keratitis (n = 3, 4%), conjunctival follicles (n = 1, 1%). Systemic side effects included post injection flu-like syndrome for 1 day (n = 7, 9%).
2012	Australia[24]	116	?	?	27 had IFNα2b alone and 89 had combined IFNα2b + retinoic acid 0·01%. Patients were prescribed IFNα2b drops 1 MIU/ml 4 times daily and retinoic acid 0·01% once every second day.	3·7% in the IFN alone 2·25% with combined treatment.	Four patients with complete response developed a mild allergic papillary conjunctivitis that settled on halving the interferon dose to 0·5 million IU drops and reducing the frequency to 3 times daily. Side effects were limited to 1 case of epithelial microcysts and 1 case of marginal keratitis.
2012	USA[25]	18	68·0	1·3:1	1 drop of 1 MIU/ml IFNα2b QID or subconjunctival 10 MIU/ml of recombinant IFN injected till the lesion balooned	None after mean 11 months follow up	Transient flu-like symptoms after IFNα2b injection (n=3). Corneal epithelial defect after 4 months of using topical IFNα2b (n=2) and Conjunctival hyperaemia (n=1). All symptoms resolved with conservative measures (e.g., acetaminophen, artificial tears, and ointments)
2012	USA[26]	20	63·0	2·3:1	Topical IFNα2b 1 million IU/mL QID continued at least 1 month beyond complete clinical resolution	5% (1 case) at 3 months follow up	Conjunctival hyperaemia (2 [10%]), follicular hypertrophy (2 [10%]), giant papillary conjunctivitis (1 [5%]), irritation (1 [5%]), corneal epithelial defect (1 [5%]), and flu-like symptoms (1 [5%]); all resolved within 1 month of medication discontinuation.
2011	USA[27]	3	41·0	2:1	IFNα2b drops (1 million units/cc) QID and IFNα2b sublesional injection (5 million units/0·5cc to 8 million units/0·8 cc)	None at 3,6, 69 months respectively	nausea and chills were minor, lasting 1 day
2010	USA[28]	15	64·0	2:1	Thrice weekly perilesional /subconjunctival 3 MIU of recombinant IFNα2b in 0·5 ml of solution. Ten eyes received concomitant	6·7% (1 eye of 15) recurred 4 months after clinical resolution	Patients were given 1000 mg acetaminophen after the injection and every 4 hours as needed to help ameliorate flulike symptoms associated with

					topical interferon therapy 4 times daily in addition to the injections. Later the injection regime was changed to received weekly until clinical disease resolution		interferon injections.
2010	USA[29]	3	63·7	All males	pegylated IFNα2β subconjunctival/perilesion injection of 1 µg/kg in the area of the lesion	1 (33·3%) recurrence after 7months follow up	No adverse effects. Acetaminophen 1000mg was given to prevent flu-like symptoms.
2010	USA[30]	33	64·4	1:2	IFNα2b drops (1 million international units (IU)/ml vs 3 million IU/ml) for 1 month after excision	9% (3 eyes) 20,21 & 28 months later	discomfort and photophobia
2008	USA[31]	29	66·7	13:1	IFNα2b drops vs no touch excision +cryotherapy and 8 had AMT too. 10 received IFNα2b 1 million IU/ml and 4 received 2 million U/ml, all 4 times per day, and 1 received 3 million IU/ml thrice daily. Those whose lesion failed to regress within 2 months of therapy crossed over to surgery. IFNα2b was continued until 4 months after clinical resolution	None a t 35·6 months	Not described
2006	Australia[32]	10	73·4	2:1	topical IFN-α2b (1 million IU/ml) four times a day until clinical resolution of the lesion or until the lesion appeared nonresponsive	None at 55 weeks follow up	Not described
2006	Australia[33]	3	69·3	1:2	IFNα2b 3Mill IU/ml every 3 days for 7 doses	None after 22·7 months follow up	Not described
2005	USA[34]	2	69·0	All males	IFNα2b drops 1 million units/ml) QID	None after 3 and 6 months follow up respectively	Not described
2004	USA[35]	7	68·7	6:1	recombinant topical IFNalpha2b drops (1 million IU/ml) 4 times daily until lesion resolution noted	2 (33·3%) patients had another recurrence of noted at 1 year and 2 months, respectively.	Not described
2002	Japan[36]	1	73·0	Female	subconj IFNα2b x2 then drops for 12wks (cryotherapy had been done twice before and failed)		Not described
1999	USA[37]	6	64·3	2:1	single subconjunctival/perilesional injection of 3 MIU/ml recombinant IFNα2b in 0·5 ml and then IFNα2b drops (1 MIU/ml) four times a day	None after 7·2 months follow up	Not described
1976	USA[38]	1	24	Male	dinitrochlorobenzene (DNCB). Initial sensitization with topical DNCB 2000µg on volar aspect of forearm. Then 30-50 µL of DNCB 2mg/ml solution in acetone applied to the tumour surface. Repeated topical treatment a week later for 5 cycles	None after 3 years follow up	Not described
**D) Immune-therapy with cyclosporin A (CSA)**							
**Year**	**Country**	**Study size**	**age (years)**	**M:F ratio**	**intervention**	**Recurrence**	**Adverse effects**
2009	Egypt[39]	10	65·0	2:1	wide surgical excision + topical cyclosporine A (0·05%) and topical mitomycin C (0·01%)	None after a mean 2 years follow up	Epithelial toxicity (punctate keratopathy) occurred in 3 eyes, ocular irritation and mild conjunctival

					QID x 12 weeks after surgery		hyperemia in 5 eyes, and lid toxicity in 2 cases during the treatment with mitomycin C
2006	Turkey[40]	2	65·0	All male	Combined CSA and MMC. CSA (0·05%) ophthalmic emulsion QID for 12 weeks, and topical MMC (0·01%) QID was combined with CSA at the second, fourth, and sixth weeks	None after 18 months follow up	In this study they lowered the concentration of MMC to 0·01% and combined it with CSA to prevent potential ocular surface side effects
**E) Chemotherapy with mitomycin C (MMC)**							
**Year**	**Country**	**Study size**	**age (years)**	**M:F ratio**	**intervention**	**Recurrence**	**Adverse effects**
2014	Australia[41]	135	69·0	3·5:1	MMC 0·04% 4 times daily for 1 week, followed by 3 weeks off treatment. IFNα2b 1 MIU/mL 4 times daily if previous treatment with MMC had failed or unable to tolerate MMC due to side effects. Again, this treatment was continued until an end point was reached. Endpoint was resolution or failure.	Overall 19 (14·1%) recurred. The mean time to recurrence was 17·2 months (range, 4 to 61 months). 14 recurrences (15·1%) were in patients treated only with MMC (n = 93), 4 recurrences (20·0%) were in patients treated with both MMC and IFNα2b (n=20), and only 1 recurrence (4·6%) was in patients treated only with IFNα2b (n= 22).	Adverse effects occurred in 76 (58·9%) patients using MMC and in 14 (30·4%) patients using IFNα2b. The most common were conjunctival hyperemia or irritation (MMC, n=63 [48·8%]; IFN, n=13 28·2%]),localized allergic or toxic reactions (defined as papillary conjunctivitis, lid swelling, or both; MMC,n = 12 [9·3%]) and punctal stenosis (MMC, n = 7 [5·4%]). There was one case of corneal erosion in each of the MMC and IFN groups. Patients occasionally had more than 1 adverse effect. There were no cases of limbal stem cell deficiency identified. Most side effects were mild and were tolerated by patients. Side effects sig- nificant enough to result in cessation of treatment before clinical resolution occurred in 12 (8·4%) of 143 eyes; all were with MMC.
2012	Turkey[42]	28	64.5	7:3	combined excision, cryotherapy, and intraoperative mitomycin-C (EXCRIM) using adjuvant 0.02 % mitomycin-C (MMC)	0% over mean 49months	7 (21 %) had delayed epithelial healing. Two of eight patients (25 %) with squamous cell carcinoma (SCC) had positive lateral margins.
2011	USA[43]	32	70·3	4:1	Excision vs Excision + MMC. intraoperative MMC use involved applying a Weck cel sponge soaked with either 0·02% or 0·04% MMC to the subconjunctival surface at the edge of the surgical excision for 1–3 minutes. Postoperatively, 1 drop of topical MMC 0·02% was given TID for 2 weeks. In most cases, 3 cycles of 2 weeks on and 2 weeks off were used with a punctal plug inserted before treatment	66·7% for excision alone, 7·7% for excision + MMC after a mean of 31·7 months follow up	Discomfort during the 2nd or 3rd treatment cycle occured in all patients
2010	Australia[44]	91	66·0	3:1	Excision + cryotherapy then MMC 0·04% (0·4 mg/ml) four times a day was used on a week-on week-off basis for two to three cycles (one cycle of treatment=1 week	12·5% after a mean 56·8 months follow up	A localised allergic reaction was seen in 23% of patients during the second or third cycle of treatment but settled rapidly on cessation of treatment in all. Of these patients, one developed a secondary levator

					on+one week off)		disinsertion ptosis requiring surgical correction. 15% of patients developed epiphora, most of which settled following simple syringing of the involved nasolacrimal system. Two patients with diffuse disease developed a corneal epithelial defect but no stromal melt. There were no incidences of severe complications such as hypotony, corneo-scleral melt or limbal stem cell failure.
2010	UK[45]	24	63·0	?	MMC as primary treatment or surgical adjuvant. 0·04% MMC four times a day for 3 weeks on, 3 weeks off, 3 weeks on, with topical steroid and lubricants throughout	67% after a mean 50 months of follow up	hyperemia, allergy, epiphora, discharge, mild keratoconjunctivitis, uveitis, corneal abrasion, corneal oedema, pyogenic granuloma, nose bleed. The most common short term complication was allergy, 64% of which occurred in the second 3-week cycle. The most common long term complication was continuing mild keratoconjunctivitis
2009	Iran[46]	17	58·7	2·4:1	2-3 alternate 7-day courses of 0·04% mitomycin-C in artificial tears. 14 patients (82·4%) received 2 courses of MMC one week apart, and only 3 patients (17·6%) received 3 MMC courses	5 (29·4%) recurrences over an mean follow up of 30·8 months	All patients reported some degrees of mild to moderate eye redness and irritation that were controlled by artificial tear, mild corticosteroid drops, and warm compress. There were no cases of scleral melting or any systemic complications
2006	Spain[47]	1	82·0	Female	Short term MMC 0·02% (2 cycles) followed by long term interferon 1 MIU/ml QID until tumour disappearance (took 75 days to disappear)	None after 1 year	no clinical evidence of limbal stem cell deficiency
2004	USA[48]	1	52·0	Female	0·02% MMC for 14 days in the first cycle, 12 days in the second cycle, and 3 days in the third cycle followed by topical interferon alfa-2b 1 x 106 U/mL for 11 days due to intolerance to MMC.	Resolution lasted 10 months. Recurrence not explicitly mentioned in	Not described
2004	Australia[49]	27	64·0	2·7:1	excised with 2mm margin + cryotherapy (where available) + Chloramphenicol and Prednefrine Forte eye drops four times daily until wound healing + at least two 1 week courses of topical MMC 0·04% four times a day, after complete epithelial healing. Each course was followed by 1 week free of MMC	None after 27 months mean follow up	5 patients developed granuloma following excision of OSSN, all of which resolved rapidly with continued topical steroid treatment. Eight patients received two courses because of allergy to MMC with redness, swelling, and significant itching, which developed late in the second course. All MMC allergy symptoms resolved rapidly following discontinuation of treatment.
2002	USA[50]	10	66·0	2·3:1	Mitomycin C 0·04% four times daily was applied for a median of three cycles	None after a median 15 months follow up	temporary local irritation, erythema, chemosis
2002	Israel[51]	5	58·4	2:3	MMC, 0·02% or 0·04%, four times daily for 14 days. Second and third identical cycles of MMC were applied at 3- to 4-week intervals. Wide excision of the conjunctiva was done 4 to 6 weeks after the final cycle of MMC	None after a mean 23·8 months follow up	1 case of conjunctival hyperaemia seen from day 8
2002	Greece[52]	7	73·8	3:4	During excision of the lesion, mitomycin-C 0·02% was applied intraoperatively for 5 minutes. In two cases, excision was combined with conjunctival limbal autograft	14% (n=1) after a mean of 16 months follow up	Not described

2002	UK[53]	11	66·7	2:9	Preoperative topical and intraoperative local mitomycin C. MMC 0·04% eye drops QID in two weekly courses preoperatively and/or a single intraoperative application of 0·4 mg/ml. 7 patients had additional limited local excision of the residual tumour mass and 1 had cryotherapy.	None after a mean of 9·5 months follow up	1 developed a reaction to the drops during the second fortnightly course of MMC drops but treatment was continued under steroid cover. 1 persistent epithelial defect following both courses of topical mitomycin C drops
2001	Germany[54]	9	?	?	mitomycin C eye drops 0·02% (MMC) after excision	11% (1 patient) 14 months after surgery and after 2 MMC-cycles.	self-limited conjunctivitis
1999	USA[55]	4	?	?	MMC 0·02% TID x2wk	None after a mean of 20 months follow up	mild discomfort, redness, photophobia, and punctate epithelial keratopathy that subsided on discontinuation of the medication
1997	UK[56]	7	?	?	one drop of MMC 0·04% QID for 7 days in alternate weeks	None after 9 months follow up	transitory ocular discomfort, conjunctival injection, tearing, photophobia, and punctate epithelial keratopathy
**F) Chemotherapy with 5-Fluorouracil (5FU)**							
**Year**	**Country**	**Study size**	**age (years)**	**M:F ratio**	**intervention**	**Recurrence**	**Adverse effects**
2015	Australia[57]	38	70·0	6:1	1% 5FU in N/saline QIDx 2wks or 0·04% MMC in N/saline x2-3 cycles each cycle lasting 1 wk followed by 1 wk drug holiday. Some pts had excision with a 2mm margin + cryotherapy	31% (10 patients) required further treatment for disease persistence or recurrence; 7 on MMC and 3 on 5FU	5-FU 1% resulted in drug-related complications in seven of 12 cases (58·3%), and included a single case of focal paracentral corneal stromal melt. MMC 0·04% resulted in transient drug related complications in 23 of 39 (59%) cases
2014	Australia[58]	153	65·5	7:3	89 on 5FU 1% QID x 2 wks and 64 on MMC 0·04% QID for 2-3 1-week cycles.	2% (n=) 2 on 5FU; 0 on MMC. Mean follow up 33·6months on 5FU and 57·9months on MMC	Not described
2011	Australia[59]	65	66·3	3:1	a single cycle of 5FU 1% in N/saline QID x 2wk	1·5% (1 case) after a median follow up 23 months	37 (57%) patients had short-term complications. Lid toxicity 32 (49·2%); epiphora 5 (7·7%); superficial keratitis 6 (9·2%); corneal epithelial defect 1 (1·5%); ectropion 1 (1·5%). Four patients were unable to complete the course of 5-FU 1% because of local toxicity
2011	Italy[60]	41	65·5	1·7:1	1% 5-FU QID x4 weeks (one course). 22 patients (53·7%) had 5-FU as a sole treatment, and 19 patients (46·3%) as adjuvant and/or debulking therapy	7·3% (3 tumours) treated with 5-FU alone recurred during a follow-up of 89·7 months	Clinical confocal microscopy showed no long-term difference between the treated eye and fellow (control) eye in: endothelial cells count, pleomorphism and polymegatism, anterior stromal keratocyte density, sub-basal nerve plexus fibre number, density, and beadings and central cornea epithelium thickness
2010	Yemen[61]	15	50·8	1:4	subconjunctival 5FU (5mg) at the end of surgery + 1% 5-FU eye drops QID x 4days then repeated at 30 day intervals x6 cycles. All had 6 months therapy	1/15 (6·7%) recurred	mild temporary local irritation
2000	USA[62]	7	74·0	All male	1% 5-FU in methylcellulose was administered four times daily for 2 to 4 days for each cycle. The number of initial treatment cycles was two to six, with the	3 recurrences (43%)	No adverse effects noted

					time between cycles being 30 to 45 days.		
2000	Italy[63]	8	70·0	3:1	1% 5FU drops alone QID x4wks without surgery or radiotherapy	1 patient (12·5%) with a mean 27 months follow up	Acute transient toxic keratoconjunctivitis was observed in all treated cases; it was controlled with topical therapy.
1995	USA[64]	6	61·0	2:1	excision +5FU or 5FU alone	1 recurred (16·6%) after 30 months	Punctate epithelial keratopathy, epithelial defect
**G) Retinoic acid**							
**Year**	**Country**	**Study size**	**age (years)**	**M:F ratio**	**intervention**	**Recurrence**	**Adverse effects**
2010	Australia[65]	1	64·0	Female	topical all-trans retinoic acid (ATRA), 0·01%, 1 eyedrop applied every second day x9mo. After there was no response from 2 intralesional injections of 3 Mill IU of recombinant IFN in 0·5 mL, 2 months apart, followed by topical interferon alfa-2b (1 Mill IU/mL) 4 times a day.	No recurrence	Not described
**H) Anti-vascular endothelial growth factor (VEGF)**							
**Year**	**Country**	**Study size**	**age (years)**	**M:F ratio**	**intervention**	**Recurrence**	**Adverse effects**
2015	Turkey[66]	6	66	2:1	topical 5mg/mL bevacizumab QID x 8 weeks	None after 6 months follow up	Not described
2014	Turkey[67]	10	60·5		bevacizumab (Avastin) 25mg/ml x 7·8wks (range 5-14) then excision biopsy+cyro+AMT	None after 6 months follow up	Not described
2009	Egypt[68]	10	45·0	2:1	subconj Avastin (bevacizumab)	20% after 12 months follow up	Not described
2009	USA[69]	4	51·5	?	Subconjunctival injections of ranibizumab 0·5 mg were given every 2 or 4 weeks if the tumour regressed or remained stable.	1 recurred (25%) and 1 did not regress (25%)	No adverse ranibizumab-related ocular or systemic side effects. Specifically, there were no local or systemic allergies and no corneal epitheliopathy, perforation, conjunctival hyperaemia, scleral thinning, uveitis orcataract formation.
**I) Radiotherapy, Thermotherapy**							
**Year**	**Country**	**Study size**	**age (years)**	**M:F ratio**	**intervention**	**Recurrence**	**Adverse effects**
2015	S. Africa[70]	69	42·0	1:1	60 Gray Sr-90 brachytherapy in four divided doses after resection with a 2 mm margin. No cryotherapy or alcohol debridement	8 (11·6%) after a median 27 months follow up	Five patients developed a dry eye, which was treated with ocular lubricants
2011	Spain[71]	1	61·0	Male	Orthovoltage was administered with direct field radiation focused on the tumor. The patient received 500 cGy in 2 sessions/week for 2 weeks (2000 cGy) and then 7 sessions 300 cGy daily (2100 cGy).	No recurrence after 1 year follow up	Not described
2009	France[72]	15	63·7	4:1	proton beam therapy and surgery	2 patients (13·2%)	Not described
2009	Australia[73]	11	60·8	1:3·7	Plaque brachytherapy. A 10- 15-mm-diameter circular Iodine-125 plaque was applied under assisted local or general anaesthesia during surgery (excised with	None after 23·4 months mean follow up	Not described

					histological confirmation using a technique involving minimal lateral clearance and avoidance of deep scleral dissection)		
1995	Germany[74]	2	58·5	All male	Brachytherapy with Ruthenium 105-applicator for radiation (after prior surgery). The applicator was fixed to the sclera adjacent to the corneal limbus. A 100 Gy radiation dose was delivered with 2mm tissue penetration.	None after 2 years follow up	Not described
1993	Austria[75]	1			local excision and brachytherapy with ruthenium-106. A total dose of 320 Gy was delivered to the tumour bed	None after 22 months	Not described
1990	Spain[76]	12			strontium-90 source on cup-shaped applicators of different sizes according to the extension of the tumor. Surface dose ranged from 60 Gy in a single treatment to 140 Gy in 7 fractions, depending on the thickness of the lesion.	1 (8·3%) recurred in a follow up period ranging from 2-15 years	Cataracts
1988	Australia[77]	146			123 were treated with a strontium-90 source, 10 with a radon "ring," and 7 with superficial X ray therapy. of the 123, 107 received 30 Gy, 14 received 40 Gy (pre 1960) and one patient each received 20 and 25 Gy incident dose	3 (2·3%)developed local recurrence	complications were very uncommon. 3 patients developed unsightly conjunctival telangiectasia, 2 patients developed a persistent scleral ulcer and 2 patients developed clinically significant cataracts.
**J) Others**							
**Year**	**Country**	**Study size**	**age (years)**	**M:F ratio**	**intervention**	**Recurrence**	**Adverse effects**
2015	USA[78]	1	64·0	Female	*Aloe vera* drops TID	None	Not described
2004	USA[79]	3	70·7	2:1	*Photodynamic therapy* 1-3 treatments of verteporfin (6 mg/m2 body surface area, intravenously). All tumours irradiated 1 min after injection using a diode laser with an emission wavelength of 695 nm, applying a light dose of 50 J/cm^2.	None after 8·6 months follow up	minimal temporary local irritation in two patients, and small conjunctival haemorrhages and mild transient chemosis in the three eyes directly after treatment. One patient had infusion-related back pain.
2002	USA[80]	1	52·0	Female	*Cidofovir drops* 6-week course of cidofovir eyedrops (2·5 mg/ml) one drop q2h x2wk then QIDx2wk then TID x2wk	None after 24 months follow up	Not described
1979	Greece[81]	9			*Urea* local application	22%	Not described

We included studies that reported recurrence as an outcome.
MIU- million international units
? means unreported or unclear

References

1. Chebbi A, Bouguila H, Boukari M, et al. [Prognosis of primary malignant tumors of the conjunctiva]. *Journal francais d'ophtalmologie* 2015; **38**(6): 477-85.
2. Li AS, Shih CY, Rosen L, Steiner A, Milman T, Udell IJ. Recurrence of Ocular Surface Squamous Neoplasia Treated With Excisional Biopsy and Cryotherapy. *American journal of ophthalmology* 2015; **160**(2): 213-9 e1.
3. Miller CV, Wolf A, Klingenstein A, et al. Clinical outcome of advanced squamous cell carcinoma of the conjunctiva. *Eye (Lond)* 2014; **28**(8): 962-7.
4. Nanji AA, Moon CS, Galor A, Sein J, Oellers P, Karp CL. Surgical versus medical treatment of ocular surface squamous neoplasia: a comparison of recurrences and complications. *Ophthalmology* 2014; **121**(5): 994-1000.
5. Crim N, Fornies-Paz ME, Monti R, Esposito E, Maccio JP, Urrets-Zavalia JA. In situ carcinoma of the conjunctiva: surgical excision associated with cryotherapy. *Clinical ophthalmology (Auckland, NZ)* 2013; **7**: 1889-93.
6. Kashkouli MB, Heirati A, Pakdel F, Kiavash V, Naseripour M, Aghamohammadi F. Long-term follow-up of invasive ocular surface squamous cell carcinoma treated with excision, cryotherapy, and topical mitomycin C. *Graefe's archive for clinical and experimental ophthalmology = Albrecht von Graefes Archiv fur klinische und experimentelle Ophthalmologie* 2012.
7. Waddell KM, Downing RG, Lucas SB, Newton R. Corneo-conjunctival carcinoma in Uganda. *Eye (Lond)* 2006; **20**(8): 893-9.
8. Peksayar G, Altan-Yaycioglu R, Onal S. Excision and cryosurgery in the treatment of conjunctival malignant epithelial tumours. *Eye (Lond)* 2003; **17**(2): 228-32.
9. Cervantes G, Rodriguez AA, Jr., Leal AG. Squamous cell carcinoma of the conjunctiva: clinicopathological features in 287 cases. *Canadian journal of ophthalmology Journal canadien d'ophtalmologie* 2002; **37**(1): 14-9; discussion 9-20.
10. Khokhar S, Soni A, SinghSethi H, Sudan R, Sony P, Pangtey MS. Combined surgery, cryotherapy, and mitomycin-C for recurrent ocular surface squamous neoplasia. *Cornea* 2002; **21**(2): 189-91.
11. McKelvie PA. Squamous cell carcinoma of the conjunctiva: a series of 26 cases. *British Journal of Ophthalmology* 2002; **86**(2): 168-73.
12. Sudesh S, Rapuano CJ, Cohen EJ, Eagle RC, Jr., Laibson PR. Surgical management of ocular surface squamous neoplasms: the experience from a cornea center. *Cornea* 2000; **19**(3): 278-83.
13. Tunc M, Char DH, Crawford B, Miller T. Intraepithelial and invasive squamous cell carcinoma of the conjunctiva: analysis of 60 cases. *British Journal of Ophthalmology* 1999; **83**(1): 98-103.
14. Tabin G, Levin S, Snibson G, Loughnan M, Taylor H. Late recurrences and the necessity for long-term follow-up in corneal and conjunctival intraepithelial neoplasia. *Ophthalmology* 1997; **104**(3): 485-92.
15. Peksayar G, Soyturk MK, Demiryont M. Long-term results of cryotherapy on malignant epithelial tumors of the conjunctiva. *American journal of ophthalmology* 1989; **107**(4): 337-40.
16. Divine RD, Anderson RL. Nitrous oxide cryotherapy for intraepithelial epithelioma of the conjunctiva. *Archives of ophthalmology* 1983; **101**(5): 782-6.
17. Palamar M, Kaya E, Egrilmez S, Akalin T, Yagci A. Amniotic membrane transplantation in surgical management of ocular surface squamous neoplasias: long-term results. *Eye (Lond)* 2014; **28**(9): 1131-5.
18. Asoklis RS, Damijonaityte A, Butkiene L, et al. Ocular surface reconstruction using amniotic membrane following excision of conjunctival and limbal tumors. *European journal of ophthalmology* 2011; **21**(5): 552-8.
19. Carvalho-Rego PR, Gomes JA, Ballalai PL, Cunha MC, Sousa LB, Erwenne CM. [Amniotic membrane for ocular surface reconstruction after conjunctival squamous cell carcinoma resection]. *Arquivos brasileiros de oftalmologia* 2008; **71**(1): 22-7.
20. Yeh LK, Lin HC, Ma DH. Amniotic membrane grafts following excision of corneal and conjunctival intraepithelial neoplasia. *Chang Gung medical journal* 2003; **26**(10): 737-44.
21. Espana EM. Amniotic membrane transplantation for reconstruction after excision of large ocular surface neoplasias. *British Journal of Ophthalmology* 2002; **86**(6): 640-5.

22. Zarei-Ghanavati S, Alizadeh R, Deng SX. Topical interferon alpha-2b for treatment of noninvasive ocular surface squamous neoplasia with 360 degrees limbal involvement. *J Ophthalmic Vis Res* 2014; **9**(4): 423-6.
23. Shields CL, Kaliki S, Kim HJ, et al. Interferon for ocular surface squamous neoplasia in 81 cases: outcomes based on the American Joint Committee on Cancer classification. *Cornea* 2013; **32**(3): 248-56.
24. Krilis M, Tsang H, Coroneo M. Treatment of conjunctival and corneal epithelial neoplasia with retinoic acid and topical interferon alfa-2b: long-term follow-up. *Ophthalmology* 2012; **119**(10): 1969-73.
25. Kim HJ, Shields CL, Shah SU, Kaliki S, Lally SE. Giant ocular surface squamous neoplasia managed with interferon alpha-2b as immunotherapy or immunoreduction. *Ophthalmology* 2012; **119**(5): 938-44.
26. Shah SU, Kaliki S, Kim HJ, Lally SE, Shields JA, Shields CL. Topical interferon alfa-2b for management of ocular surface squamous neoplasia in 23 cases: outcomes based on american joint committee on cancer classification. *Archives of ophthalmology* 2012; **130**(2): 159-64.
27. Shields CL, Kancherla S, Bianciotto CG, Lally SE, Shields JA. Ocular surface squamous neoplasia (squamous cell carcinoma) of the socket: management of extensive tumors with interferon. *Ophthalmic plastic and reconstructive surgery* 2011; **27**(4): 247-50.
28. Karp CL, Galor A, Chhabra S, Barnes SD, Alfonso EC. Subconjunctival/perilesional recombinant interferon alpha2b for ocular surface squamous neoplasia: a 10-year review. *Ophthalmology* 2010; **117**(12): 2241-6.
29. Karp CL, Galor A, Lee Y, Yoo SH. Pegylated interferon alpha 2b for treatment of ocular surface squamous neoplasia: a pilot study. *Ocular immunology and inflammation* 2010; **18**(4): 254-60.
30. Galor A, Karp CL, Chhabra S, Barnes S, Alfonso EC. Topical interferon alpha 2b eye-drops for treatment of ocular surface squamous neoplasia: a dose comparison study. *The British journal of ophthalmology* 2010; **94**(5): 551-4.
31. Sturges A, Butt AL, Lai JE, Chodosh J. Topical interferon or surgical excision for the management of primary ocular surface squamous neoplasia. *Ophthalmology* 2008; **115**(8): 1297-302, 302 e1.
32. Holcombe DJ, Lee GA. Topical Interferon Alfa-2b for the Treatment of Recalcitrant Ocular Surface Squamous Neoplasia. *American journal of ophthalmology* 2006; **142**(4): 568-71.e3.
33. Nemet AY, Sharma V, Benger R. Interferon alpha 2b treatment for residual ocular surface squamous neoplasia unresponsive to excision, cryotherapy and mitomycin-C. *Clin Experiment Ophthalmol* 2006; **34**(4): 375-7.
34. Esquenazi S, Fry CL, Holley E. Treatment of biopsy proved conjunctival intraepithelial neoplasia with topical interferon alfa-2b. *The British journal of ophthalmology* 2005; **89**(9): 1221.
35. Boehm MD, Huang AJ. Treatment of recurrent corneal and conjunctival intraepithelial neoplasia with topical interferon alfa 2b. *Ophthalmology* 2004; **111**(9): 1755-61.
36. Kobayashi A, Yoshita T, Uchiyama K, et al. Successful management of conjunctival intraepithelial neoplasia by interferon alpha-2b. *Japanese journal of ophthalmology* 2002; **46**(2): 215-7.
37. Vann RR, Karp CL. Perilesional and topical interferon alfa-2b for conjunctival and corneal neoplasia1. *Ophthalmology* 1999; **106**(1): 91-7.
38. Ferry AP, Meltzer MA, Taub RN. Immunotherapy with dinitrochlorobenzene (DNCB) for recurrent squamous cell tumor of conjunctiva. *Transactions of the American Ophthalmological Society* 1976; **74**: 154-71.
39. Zaki AA, Farid SF. Management of intraepithelial and invasive neoplasia of the cornea and conjunctiva: a long-term follow up. *Cornea* 2009; **28**(9): 986-8.
40. Tunc M, Erbilen E. Topical cyclosporine-a combined with mitomycin C for conjunctival and corneal squamous cell carcinoma. *American journal of ophthalmology* 2006; **142**(4): 673-5.
41. Besley J, Pappalardo J, Lee GA, Hirst LW, Vincent SJ. Risk factors for ocular surface squamous neoplasia recurrence after treatment with topical mitomycin C and interferon alpha-2b. *American journal of ophthalmology* 2014; **157**(2): 287-93 e2.

42. Sarici AM, Arvas S, Pazarli H. Combined excision, cryotherapy, and intraoperative mitomycin C (EXCRIM) for localized intraepithelial and squamous cell carcinoma of the conjunctiva. *Graefe's archive for clinical and experimental ophthalmology = Albrecht von Graefes Archiv fur klinische und experimentelle Ophthalmologie* 2013; **251**(9): 2201-4.
43. Birkholz ES, Goins KM, Sutphin JE, Kitzmann AS, Wagoner MD. Treatment of ocular surface squamous cell intraepithelial neoplasia with and without mitomycin C. *Cornea* 2011; **30**(1): 37-41.
44. Gupta A, Muecke J. Treatment of ocular surface squamous neoplasia with Mitomycin C. *The British journal of ophthalmology* 2010; **94**(5): 555-8.
45. Russell HC, Chadha V, Lockington D, Kemp EG. Topical mitomycin C chemotherapy in the management of ocular surface neoplasia: a 10-year review of treatment outcomes and complications. *The British journal of ophthalmology* 2010; **94**(10): 1316-21.
46. Rahimi F, Alipour F, Ghazizadeh Hashemi H, Hashemian MN, Mehrdad R. Topical mitomycin-C for treatment of partially-excised ocular surface squamous neoplasia. *Archives of Iranian medicine* 2009; **12**(1): 55-9.
47. Huerva V, Mateo AJ, Mangues I, Jurjo C. Short-term mitomycin C followed by long-term interferon alpha2beta for conjunctiva-cornea intraepithelial neoplasia. *Cornea* 2006; **25**(10): 1220-3.
48. Di Pascuale MA, Espana EM, Tseng SC. A case of conjunctiva-cornea intraepithelial neoplasia successfully treated with topical mitomycin C and interferon alfa-2b in cycles. *Cornea* 2004; **23**(1): 89-92.
49. Chen C, Louis D, Dodd T, Muecke J. Mitomycin C as an adjunct in the treatment of localised ocular surface squamous neoplasia. *The British journal of ophthalmology* 2004; **88**(1): 17-8.
50. Shields CL, Naseripour M, Shields JA. Topical mitomycin C for extensive, recurrent conjunctival-corneal squamous cell carcinoma. *American journal of ophthalmology* 2002; **133**(5): 601-6.
51. Frucht-Pery J, Rozenman Y, Pe'er J. Topical mitomycin-C for partially excised conjunctival squamous cell carcinoma. *Ophthalmology* 2002; **109**(3): 548-52.
52. Siganos CS, Kozobolis VP, Christodoulakis EV. The intraoperative use of mitomycin-C in excision of ocular surface neoplasia with or without limbal autograft transplantation. *Cornea* 2002; **21**(1): 12-6.
53. Kemp EG, Harnett AN, Chatterjee S. Preoperative topical and intraoperative local mitomycin C adjuvant therapy in the management of ocular surface neoplasias. *The British journal of ophthalmology* 2002; **86**(1): 31-4.
54. Cartsburg O, Kersten A, Sundmacher R, Nadjari B, Pomjanski N, Bocking A. [Treatment of 9 squamous epithelial carcinoma in situ lesions of the conjunctiva (CIN) with mitomycin C eyedrops in cytological and DNA image cytometric control]. *Klinische Monatsblatter fur Augenheilkunde* 2001; **218**(6): 429-34.
55. Akpek EK, Ertoy D, Kalayci D, Hasiripi H. Postoperative topical mitomycin C in conjunctival squamous cell neoplasia. *Cornea* 1999; **18**(1): 59-62.
56. Wilson MW, Hungerford JL, George SM, Madreperla SA. Topical mitomycin C for the treatment of conjunctival and corneal epithelial dysplasia and neoplasia. *American journal of ophthalmology* 1997; **124**(3): 303-11.
57. Rudkin AK, Dempster L, Muecke JS. Management of diffuse ocular surface squamous neoplasia: efficacy and complications of topical chemotherapy. *Clinical & Experimental Ophthalmology* 2015; **43**(1): 20-5.
58. Bahrami B, Greenwell T, Muecke JS. Long-term outcomes after adjunctive topical 5-flurouracil or mitomycin C for the treatment of surgically excised, localized ocular surface squamous neoplasia. *Clin Experiment Ophthalmol* 2014; **42**(4): 317-22.
59. Rudkin AK, Muecke JS. Adjuvant 5-fluorouracil in the treatment of localised ocular surface squamous neoplasia. *The British journal of ophthalmology* 2011; **95**(7): 947-50.
60. Parrozzani R, Lazzarini D, Alemany-Rubio E, Urban F, Midena E. Topical 1% 5-fluorouracil in ocular surface squamous neoplasia: a long-term safety study. *The British journal of ophthalmology* 2011; **95**(3): 355-9.
61. Al-Barrag A, Al-Shaer M, Al-Matary N, Al-Hamdani M. 5-Fluorouracil for the treatment of intraepithelial neoplasia and squamous cell carcinoma of the conjunctiva, and cornea. *Clinical ophthalmology (Auckland, NZ)* 2010; **4**: 801-8.

62. Yeatts RP, Engelbrecht NE, Curry CD, Ford JG, Walter KA. 5-Fluorouracil for the treatment of intraepithelial neoplasia of the conjunctiva and cornea. *Ophthalmology* 2000; **107**(12): 2190-5.
63. Midena E. Treatment of conjunctival squamous cell carcinoma with topical 5-fluorouracil. *British Journal of Ophthalmology* 2000; **84**(3): 268-72.
64. Yeatts RP, Ford JG, Stanton CA, Reed JW. Topical 5-fluorouracil in treating epithelial neoplasia of the conjunctiva and cornea. *Ophthalmology* 1995; **102**(9): 1338-44.
65. Skippen B, Tsang HH, Assaad NN, Coroneo MT. Rapid response of refractory ocular surface dysplasia to combination treatment with topical all-trans retinoic acid and interferon alfa-2b. *Archives of ophthalmology* 2010; **128**(10): 1368-9.
66. Asena L, Dursun Altinors D. Topical Bevacizumab for the Treatment of Ocular Surface Squamous Neoplasia. *Journal of ocular pharmacology and therapeutics : the official journal of the Association for Ocular Pharmacology and Therapeutics* 2015.
67. Ozcan AA, Ciloglu E, Esen E, Simdivar GH. Use of topical bevacizumab for conjunctival intraepithelial neoplasia. *Cornea* 2014; **33**(11): 1205-9.
68. Zaki AA, Fouad H, Emera S, Labibi H. Subconjunctival Anti VEGF for Conjunctival Intraepithelial and Invasive Neoplasia. *Australian Journal of Basic and Applied Sciences,* 2009; **3**(4): 3186-9.
69. Teng CC, Chin KJ, Finger PT. Subconjunctival ranibizumab for squamous cell carcinoma of the conjunctiva with corneal extension. *The British journal of ophthalmology* 2009; **93**(6): 837-8.
70. Lecuona K, Stannard C, Hart G, et al. The treatment of carcinoma in situ and squamous cell carcinoma of the conjunctiva with fractionated strontium-90 radiation in a population with a high prevalence of HIV. *The British journal of ophthalmology* 2015; **99**(9): 1158-61.
71. Sanchez-Perez JL, Fuentes-Sanchez C, Acosta-Acosta B. Conjunctival-corneal intraepithelial neoplasia (Bowen disease) treated with orthovoltage. *Cornea* 2011; **30**(4): 474-6.
72. Caujolle JP, Maschi C, Chauvel P, Herault J, Gastaud P. [Surgery and additional protontherapy for treatment of invasive and recurrent squamous cell carcinomas: technique and preliminary results]. *Journal francais d'ophtalmologie* 2009; **32**(10): 707-14.
73. Walsh-Conway N, Conway RM. Plaque brachytherapy for the management of ocular surface malignancies with corneoscleral invasion. *Clin Experiment Ophthalmol* 2009; **37**(6): 577-83.
74. Haberle H, Pham DT, Scholman HJ, Wollensak J. [Ruthenium 105-applicator for radiation treatment of carcinoma in situ of the cornea and conjunctiva]. *Der Ophthalmologe : Zeitschrift der Deutschen Ophthalmologischen Gesellschaft* 1995; **92**(6): 866-9.
75. Zehetmayer M, Menapace R, Kulnig W. Combined local excision and brachytherapy with ruthenium-106 in the treatment of epibulbar malignancies. *Ophthalmologica Journal international d'ophtalmologie International journal of ophthalmology Zeitschrift fur Augenheilkunde* 1993; **207**(3): 133-9.
76. Cerezo L, Otero J, Aragon G, et al. Conjunctival intraepithelial and invasive squamous cell carcinomas treated with strontium-90. *Radiotherapy and oncology : journal of the European Society for Therapeutic Radiology and Oncology* 1990; **17**(3): 191-7.
77. Kearsley JH, Fitchew RS, Taylor RG. Adjunctive radiotherapy with strontium-90 in the treatment of conjunctival squamous cell carcinoma. *International journal of radiation oncology, biology, physics* 1988; **14**(3): 435-43.
78. Damani MR, Shah AR, Karp CL, Orlin SE. Treatment of ocular surface squamous neoplasia with topical Aloe vera drops. *Cornea* 2015; **34**(1): 87-9.
79. Barbazetto IA, Lee TC, Abramson DH. Treatment of conjunctival squamous cell carcinoma with photodynamic therapy. *American journal of ophthalmology* 2004; **138**(2): 183-9.
80. Sherman MD, Feldman KA, Farahmand SM, Margolis TP. Treatment of conjunctival squamous cell carcinoma with topical cidofovir. *American journal of ophthalmology* 2002; **134**(3): 432-3.
81. Danopoulos ED, Danopoulou IE, Liarikos SB, Merkuris KM. Effects of urea treatment in malignancies of the conjunctiva and cornea. *Ophthalmologica Journal international d'ophtalmologie International journal of ophthalmology Zeitschrift fur Augenheilkunde* 1979; **178**(4): 198-203.
